# Altered Brain Structural Networks in Patients with Brain Arteriovenous Malformations Located in Broca's Area

**DOI:** 10.1155/2020/8886803

**Published:** 2020-10-24

**Authors:** Maogui Li, Pengjun Jiang, Jun Wu, Rui Guo, Xiaofeng Deng, Yong Cao, Shuo Wang

**Affiliations:** ^1^Department of Neurosurgery, Beijing Tiantan Hospital, Capital Medical University, Beijing, China; ^2^China National Clinical Research Center for Neurological Diseases, Beijing, China; ^3^Center of Stroke, Beijing Institute for Brain Disorders, Beijing, China; ^4^Beijing Key Laboratory of Translational Medicine for Cerebrovascular Diseases, Beijing, China

## Abstract

Focal brain lesions, such as stroke and tumors, can lead to remote structural alterations across the whole-brain networks. Brain arteriovenous malformations (AVMs), usually presumed to be congenital, often result in tissue degeneration and functional displacement of the perifocal areas, but it remains unclear whether AVMs may produce long-range effects upon the whole-brain white matter organization. In this study, we used diffusion tensor imaging and graph theory methods to investigate the alterations of brain structural networks in 14 patients with AVMs in the presumed Broca's area, compared to 27 normal controls. Weighted brain structural networks were constructed based on deterministic tractography. We compared the topological properties and network connectivity between patients and normal controls. Functional magnetic resonance imaging revealed contralateral reorganization of Broca's area in five (35.7%) patients. Compared to normal controls, the patients exhibited preserved small-worldness of brain structural networks. However, AVM patients exhibited significantly decreased global efficiency (*p* = 0.004) and clustering coefficient (*p* = 0.014), along with decreased corresponding nodal properties in some remote brain regions (*p* < 0.05, family-wise error corrected). Furthermore, structural connectivity was reduced in the right perisylvian regions but enhanced in the perifocal areas (*p* < 0.05). The vulnerability of the left supramarginal gyrus was significantly increased (*p* = 0.039, corrected), and the bilateral putamina were added as hubs in the AVM patients. These alterations provide evidence for the long-range effects of AVMs on brain white matter networks. Our preliminary findings contribute extra insights into the understanding of brain plasticity and pathological state in patients with AVMs.

## 1. Introduction

Brain arteriovenous malformations (AVMs) are commonly presumed to be congenital lesions with the pathology of direct connection between arteries and veins, resulting in arteriovenous shunting of blood [1, 2]. The local hemodynamic abnormalities associated with arteriovenous shunts may lead to pathological changes in the surrounding brain parenchyma, which are thought to be the causes of seizures and neurological dysfunction [1, 3, 4]. Besides, it has been found that the local damage may cause cortical functional reorganization, mostly in those patients who have lesions right in or close to the presumed eloquent areas [5–8]. Most of the current studies of AVMs focus on the structural and functional changes of the perifocal areas. However, the high incidence of cognitive decline reported in AVM patients cannot be fully explained by these local effects [9]. In other words, the potential long-range effects of AVMs might have been overlooked. Recent advancement of brain connectome analyses has provided new insights into the interactions between brain regions at the whole-brain level. Based on the growing number of results from studies of functional and structural connectivity, it has been recognized that focal brain lesions, such as stroke and tumors, can affect not only the surrounding tissue but also the remote brain regions across the whole-brain networks [10–13]. A recent resting-state network study found the disruption of normal anticorrelation between the default mode network and the attentional control network in AVM patients, contributing to the discussion on the remote effects of AVMs [14].

The graph theory analysis provides an outstanding framework for studying the complex brain networks [15, 16]. Recently, using diffusion tensor imaging (DTI) and graph theory methods, disrupted topological organization of the white matter networks has been revealed in various psychiatric and degenerative disorders [17, 18]. Several studies have demonstrated the altered brain structural networks in patients with stroke and the correlations between the topological properties and functional outcomes [19–21]. In brain tumor patients, a study showed notable alterations in topological properties of brain structural networks compared to healthy subjects, which were considered evidence of compensatory structural plasticity [22]. By comparison with stroke and tumors, AVMs produce effects on the brain from much earlier life due to the congenital and chronic nature course. Since structural connectivity is thought to be the physical substrate of functional interactions across the brain [23, 24], analysis of altered structural networks will help in understanding the brain plasticity associated with AVMs.

We hypothesized that AVMs not only cause brain reorganization of the perifocal areas but also have long-range effects on the whole-brain white matter organization. In this study, we selectively included patients with AVMs located in the presumed Broca's area, in order to investigate the alterations in topological properties and network connectivity of brain structural networks.

## 2. Materials and Methods

### 2.1. Subjects

This study was approved by the institutional review board of Beijing Tiantan Hospital, Capital Medical University. The participants provided their written informed consent to participate in the study. From January 2015 to June 2019, patients with AVMs in the left frontal lobe were screened out from our clinical trial database (ClinicalTrials.gov, identifier: NCT02868008). The inclusion criteria were as follows: (1) patients with lesions located in the left frontal lobe, wholly or partially overlying the presumed Broca's area (the classical Brodmann areas 44 and 45, i.e., the pars opercularis and pars triangularis of the inferior frontal gyrus); (2) patients with no history of AVM rupture; (3) patients with no apparent perifocal edema on T2-weighted images; (4) patients with no apparent space-occupying effect (e.g., space-occupying dilated draining veins); and (5) patients with no other neurological or psychiatric disorders.

Fourteen patients were included in this study, of whom eight (57.1%) were female and six (42.9%) were male, with ages ranging from 15 to 38 years (mean ± SD, 27.4 ± 7.0 years). All patients were native Chinese speakers and right-handed, and no patient showed language deficits before the participation. Meanwhile, 27 right-handed normal controls with no history of neurological or psychiatric disorders were recruited, of whom 15 (55.6%) were female, with ages ranging from 19 to 40 years (mean ± SD, 25.0 ± 4.0 years).

### 2.2. Image Acquisition

All subjects had undergone T1-weighted and T2-weighted imaging. Furthermore, the AVM patients went through DTI and language fMRI examination, while the normal controls only underwent DTI scanning. MRI data was acquired with a 3.0 T system (MAGNETOM Trio, Siemens Healthineers, Erlangen, Germany) with the following parameters:

1. T1-weighted anatomical images were acquired with a 3D MP-RAGE sequence, with TR = 2300 ms, TE = 2.98 ms, flip angle = 9°, voxel size = 1 × 1 × 1 mm, slice thickness = 1 mm, FOV = 240 × 256 mm, matrix size = 240 × 256, and sagittal slices = 176
2. Diffusion tensor images were acquired with an EPI sequence, with *b*‐value = 1000 along 30 diffusion directions, TR = 6800 ms, TE = 93 ms, flip angle = 120°, voxel size = 1.8 × 1.8 × 2.8 mm, slice thickness = 2.8 mm, FOV = 230 × 230 mm, matrix size = 128 × 128, slice number = 50, double averaging, and parallel acquisition (GRAPPA factor = 2)
3. Functional images were acquired with an EPI sequence, with TR = 3000 ms, TE = 30 ms, flip angle = 90°, voxel size = 3.3 × 3.3 × 4 mm, slice thickness = 4 mm, FOV = 210 × 210 mm, matrix size = 64 × 64, and slice number = 30

### 2.3. Lesion Mapping

The boundaries of the AVMs were manually drawn on the anatomical T1-weighted images slice by slice with the MRIcron software (https://people.cas.sc.edu/rorden/mricron/). Next, using the clinical toolbox (https://www.nitrc.org/projects/clinicaltbx/) on SPM12, the lesion masks were normalized with the individual's T1-weighted images into the Montreal Neurological Institute (MNI) space and were then overlapped on an MNI template for display.

### 2.4. DTI Processing and Network Construction

All processes were following the accepted pipeline. Structural segmentation was performed with the FreeSurfer software package (http://surfer.nmr.mgh.harvard.edu/). In order to overcome the possible signal loss and structural distortions of the lesioned brain, we employed the enantiomorphic filling method using the clinical toolbox on SPM12 [25, 26]. The steps were as follows. First, for each patient, the mirrored T1-weighted image was created and coregistered to the native T1-weighted image. Next, a chimeric anatomical image was generated based on the native T1-weighted image with the lesion area replaced by tissue from the homologous region of the intact contralateral hemisphere (smoothed lesion mask was used for inserting tissue). Then, the enantiomorphic filling anatomical images instead of the original T1-weighted images were processed. This fix enabled FreeSurfer to properly trace the cortical surface and the subcortical structures [27]. The cerebral cortex was automatically parcellated into 68 cortical areas according to the Desikan-Killiany atlas. The 14 automatically segmented subcortical gray matter volumes were added to the cortical parcellation [28, 29]. Next, the voxels of the resulting segmentation that correspond to the lesion area were excluded by the normalized lesion mask. Careful visual inspection and possibly additional manual editing were required to ensure accuracy. Finally, the 82 regions of interest (ROIs) (41 ROIs for each hemisphere, Table 1) were warped into the individual's native diffusion space with the nearest neighbor resampling for network analysis.

Diffusion images were processed by using the FSL diffusion toolbox (http://www.fmrib.ox.ac.uk/fsl) and the PANDA pipeline toolbox (https://www.nitrc.org/projects/panda/). Eddy currents and head motion artifacts were corrected. A whole-brain deterministic tractography was performed by using the Fiber Assignment by Continuous Tracking (FACT) algorithm. Fiber tracking was stopped at voxels with FA less than 0.15, and the curvature angle threshold was set to 45°.

The whole-brain structural networks were represented as an adjacent matrix. The parcellated 82 gray matter ROIs were used as nodes, and the edges were defined as the white matter fibers connecting each pair of nodes. If the number of streamlines between two nodes was less than three, the connection would be set to zero in order to reduce possible spurious connections. The connectivity strength (*w*_*ij*_) between a pair of nodes *i* and *j* was the number of streamlines with a correction with the distance travelled by the fibers and the volumes of ROI *i* and *j*. Thus, a weighted 82 × 82 structural connectivity matrix was generated for each subject.

### 2.5. Network Graph Analysis

Based on graph theory, the topological properties of the weighted networks were analyzed at global and nodal level using the brainGraph toolbox available in R (https://github.com/cwatson/brainGraph). The nodal topological properties computed included the connectivity strength, nodal efficiency, local clustering coefficient, betweenness centrality, and vulnerability. The global topological properties computed included the global efficiency, local efficiency, clustering coefficient, characteristic path length, and small-worldness.

#### 2.5.1. Nodal Topological Properties

The connectivity strength (*S*_*i*_) of node *i* is the sum of the weights of all its connections:

(1) $$\begin{array}{c} S_{i}=\underset{j\in N}{\sum }w_{ij}, \end{array}$$

where *w*_*ij*_ is the connectivity weight between nodes *i* and *j* and *N* is the set of all 82 nodes of the network.

The nodal efficiency (*E*_nod_) measures the ability of node *i* to exchange information with the remaining nodes in the network:

(2) $$\begin{array}{c} E_{\text{nod},i}=\frac{1}{N-1}\underset{j\in N}{\sum }\frac{1}{L_{ij}}, \end{array}$$

where *N* is the number of nodes within the network and *L*_*ij*_ was the length of the shortest weighted path between nodes *i* and *j*.

The local clustering coefficient (*C*_*i*_) of node *i* describes how strong the node and its neighbors tend to cluster as a local network:

(3) $$\begin{array}{c} C_{i}=\underset{j,h\in N}{\sum }\frac{{\left(w_{ij}w_{ih}w_{jh}\right)}^{1/3}}{k_{i}\left(k_{i}-1\right)}, \end{array}$$

where *k*_*i*_ is the number of edges connected to node *i* and *w*_*ij*_, *w*_*ih*_, and *w*_*jh*_ are the weights between node *i* and its neighbor nodes *j* and *h* and the weight between *j* and *h*.

The betweenness centrality (*B*_*i*_) quantifies the centrality of node *i*, which is the fraction of all the shortest paths in the network that contain *i*:

(4) $$\begin{array}{c} B_{i}=\frac{1}{\left(N-1\right)\left(N-2\right)}\underset{j,h\in N}{\sum }\frac{\delta_{jh}\left(i\right)}{\delta_{jh}}, \end{array}$$

where *δ*_*jh*_(*i*) is number of the shortest paths between nodes *j* and *h* that pass through node *i*. In this study, node *i* was defined as a hub, if *B*_*i*_ was at least one standard deviation (SD) greater than the average betweenness centrality of the network.

The vulnerability (*V*_*i*_) reflects the influence of node *i* on the network efficiency, which is defined as the proportional drop in global efficiency when *i* and its connections are removed from the network:

(5) $$\begin{array}{c} V_{i}=\frac{E_{\text{glob}}-E_{\text{glob}}^{i}}{E_{\text{glob}}}, \end{array}$$

where *E*_glob_^*i*^ is the global efficiency after removing node *i*.

#### 2.5.2. Global Topological Properties

The global efficiency (*E*_glob_) measures how efficient the information is exchanged across the whole network, which is the average of the nodal efficiency values of all nodes in the network:

(6) $$\begin{array}{c} E_{\text{glob}}=\frac{1}{N}\underset{i\in N}{\sum }E_{\text{nod},i}. \end{array}$$

The local efficiency (E_*loc*_) measures the average capacity for information transfer within a local network:

(7) $$\begin{array}{c} E_{\text{loc}}=\frac{1}{N}\underset{i\in N}{\sum }E_{\text{glob},i}, \end{array}$$

where *E*_glob,*i*_ is the global efficiency of the subgraph consisting of the neighbors of node *i*.

The global clustering coefficient (*C*_*p*_) measures the degree to which the nodes in the network tend to cluster, which is the average of the local clustering coefficient values of all nodes:

(8) $$\begin{array}{c} C_{p}=\frac{1}{N}\underset{i\in N}{\sum }C_{i}. \end{array}$$

The characteristic path length (*L*_*p*_) is the harmonic mean of the shortest path between each pair of nodes in the network, which reflects how well the entire network is connected for information exchange. *L*_*p*_ equals to the inverse of *E*_glob_:

(9) $$\begin{array}{c} L_{p}=\frac{1}{E_{\text{glob}}}. \end{array}$$

The small-worldness (*σ*) is defined as the ratio between the normalized clustering coefficient (*γ*) and the normalized characteristic path length (*λ*):

(10) $$\begin{array}{c} \gamma =\frac{C_{p}}{C_{p}^{\mathrm{rand}}},\\ \lambda =\frac{L_{p}}{L_{p}^{\mathrm{rand}}},\\ \sigma =\frac{\gamma }{\lambda }, \end{array}$$

where *C*_*p*_^rand^ and *L*_*p*_^rand^ represent the average of the global clustering coefficient and characteristic path length of 100 matched random networks with the same numbers of nodes, edges, and degree distributions as the real network. A network is considered a small-world network if *σ* > 1.

### 2.6. fMRI Analysis

We performed a verb generation task to test the hemispheric lateralization of the frontal language area. It has been proven that verb generation exhibits strong reliability and robustness for measuring laterality, especially in the frontal areas [30]. The task was block-designed consisting of eight 21-second baseline blocks and eight 15-second task blocks. In a task period, five common concrete nouns were presented to the patient. The patients were instructed to generate a verb associated with each noun silently (e.g., bike = ride). In the rest period, the patients were asked to fix their gaze on a pound sign.

Functional images were processed with the widely accepted pipeline of SPM12 (https://www.fil.ion.ucl.ac.uk/spm/software/spm12/) on MATLAB 2017b (The MathWorks Inc., Natick, USA). We employed the enantiomorphic filling and unified segmentation approach for normalization in image preprocessing [25, 31]. Activation maps were generated at the threshold of *p* < 0.001 with cluster size ≥ 10 voxels. The frontal ROI was defined as the pars opercularis and pars triangularis of the inferior frontal gyrus and the dorsolateral prefrontal cortex (the classical Brodmann areas 44, 45, 46, and 9). The laterality index (LI, ranging from -1 to 1) was calculated as LI = (*L* − *R*)/(*L* + *R*), where *L* and *R* represented the number of voxels activated in the ROIs of the left and right hemisphere. A patient was classified as typically left-lateralized if LI ≥ 0.2 and atypically lateralized if LI < 0.2.

### 2.7. Statistical Analysis

Statistical analysis was performed with the brainGraph toolbox in R (R Core Team, 2019). BrainNet Viewer (http://www.nitrc.org/projects/bnv/) was used to visualize the results. The difference in global topological properties (*E*_glob_, *E*_loc_, *C*_*p*_, *L*_*p*_, and *σ*) between patients and normal controls was tested using permutation inference for a general linear model (GLM), with age and sex as covariates. The one-tailed *p* values were computed using 10000 permutations, and the significance threshold was set at *p* < 0.05. Between-group comparisons of the nodal topological properties (*S*_*i*_, *E*_nod,*i*_, *C*_*i*_, *B*_*i*_, and *V*_*i*_) of each region were also performed using permutation inference for GLM with age and sex as covariates and were corrected for multiple comparisons with the family-wise error (FWE). The one-tailed *p* values were computed using 10000 permutations, and a corrected *p* value less than 0.05 was considered significant.

The network-based statistic (NBS) was used to identify the altered structural connectivity between the patients and normal controls [32]. Briefly, the NBS method is to identify significantly altered connected components, which can provide greater statistical power, compared to individually testing each independent connection. An initial threshold is needed for determining the clusters of connections. Since the choice of the initial threshold is arbitrary, a strict initial *p* < 0.005 and a less strict initial *p* < 0.05 were used in this study. Permutation analysis is then used to evaluate the null hypothesis on the level of connected clusters. We performed 10000 permutations, and a threshold of *p* < 0.05 was used to identify the final components. Age and sex were included as covariates.

## 3. Results

**Figure 1:**
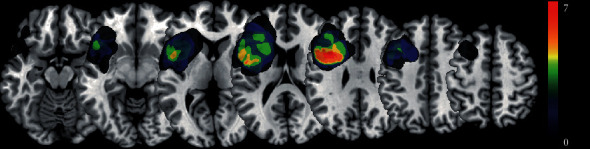
Lesion overlay of the 14 patients. Colors represent the number of patients with a lesion in the area. Warmer colors indicate a greater overlap.

The demographics and the number of whole-brain streamlines of normal controls and AVM patients are presented in Table 2. No significant difference was found in age, sex, and the number of streamlines between the two groups. The average diameter of the AVMs was 3.6 ± 1.3 cm.  Figure 1 shows the lesion overlay of all the patients.

### 3.1. Global Topological Properties of Brain Structural Networks

**Figure 2:**
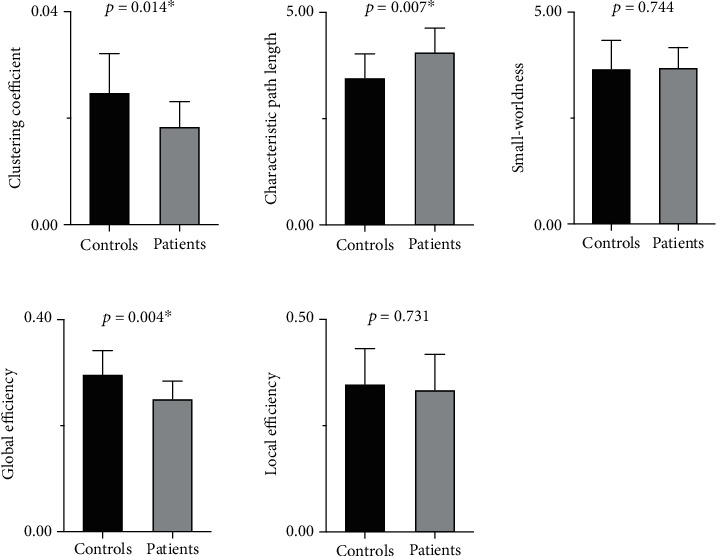
Comparison of the global topological properties of brain structural networks between patients and normal controls.

The global topological properties of brain structural networks of the normal controls and patients are presented in Figure 2. Both normal controls and patients exhibited small-world organization (*σ* > 1) in brain structural networks. No significant between-group difference was found in the small-worldness (*p* = 0.744) or in the local efficiency (*p* = 0.731). However, AVM patients showed significantly greater characteristic path length (*p* = 0.007) and lower clustering coefficient (*p* = 0.014). In addition, the global efficiency was significantly decreased in the patients (*p* = 0.004).

### 3.2. Nodal Topological Properties of Brain Structural Networks

**Figure 3:**
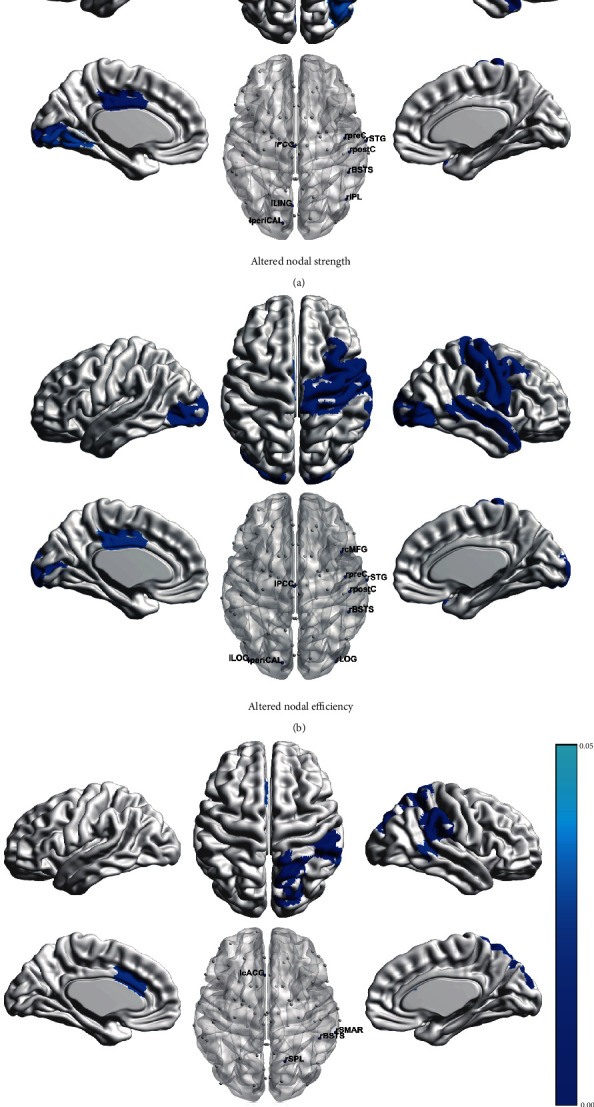
Differences in nodal topological properties of brain structural networks between normal controls and patients. (a) Regions with decreased nodal strength in patients. (b) Regions with decreased nodal efficiency in patients. (c) Regions with decreased local clustering coefficient in patients. The differences survived critical FWE correction.

Brain regions with significant between-group difference (corrected *p* < 0.05) in nodal properties were identified. A number of regions were found with significantly lower nodal strength, nodal efficiency, and local clustering coefficient in AVM patients relative to normal controls (Figure 3). Decreased nodal strength was observed in the lingual gyrus (LING), pericalcarine cortex (periCAL), and posterior cingulate gyrus (PCG) in the left hemisphere and the precentral gyrus (preC), postcentral gyrus (postC), inferior parietal lobule, bank of the superior temporal sulcus (BSTS), and superior temporal gyrus (STG) in the right hemisphere (Figure 3(a)). Decreased nodal efficiency was found in the lateral occipital gyrus (LOG), periCAL, and PCG in the left hemisphere and the caudal middle frontal gyrus, preC, postC, BSTS, STG, and LOG in the right hemisphere (Figure 3(b)). Decreased local clustering coefficient was found in the right BSTS, right superior parietal lobule, right supramarginal gyrus (SMAR), and left caudal anterior cingulate gyrus (Figure 3(c)). Furthermore, a significant increase in vulnerability was found exclusively in the left SMAR in the patients (*p* = 0.039, corrected). No significant difference was found in the nodal betweenness centrality.

### 3.3. Hub Region Distribution

**Figure 4:**
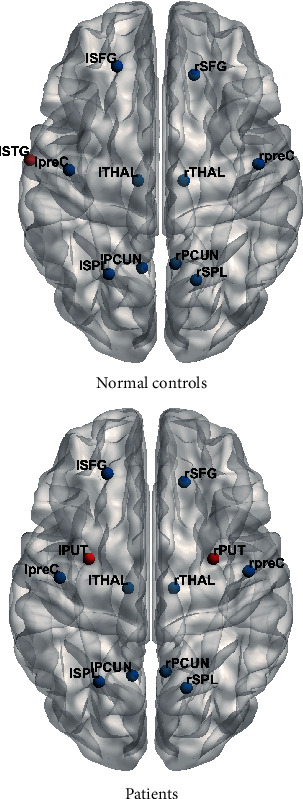
Hub region distribution in patients and normal controls. The nodes color-coded in blue were shared by the two groups, while the nodes color-coded in red were not.

In normal controls, we identified 11 hub regions, of which nine were cortical and two were subcortical (Figure 4). In AVM patients, we identified 12 hub regions, of which eight were cortical and four were subcortical. The mean betweenness centrality and mean strength of the hub regions are shown in Table 3. Ten regions were shared by the two groups. However, the left STG was identified as a hub for normal controls but not for the patients, while the bilateral putamina were identified as hubs for the patients but not for the controls.

### 3.4. Network-Based Statistic Results

**Figure 5:**
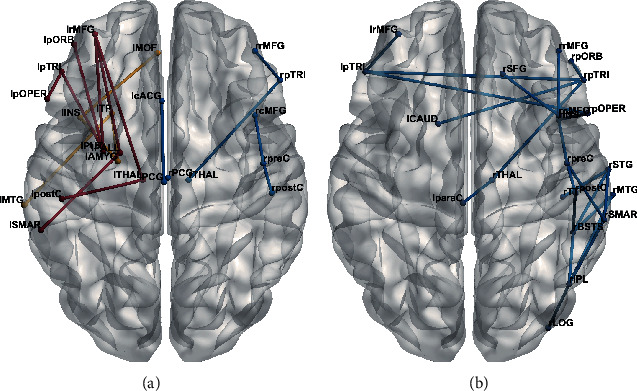
Altered structural connectivity derived from the network-based statistic. (a) An initial *p* < 0.005 resulted in three subnetworks color-coded in blue exhibiting decreased structural connectivity and two subnetworks color-coded in red/orange exhibiting enhanced structural connectivity in patients relative to normal controls. (b) An initial *p* < 0.05 resulted in a subnetwork color-coded in blue exhibiting decreased structural connectivity in patients relative to normal controls.

Using an initial significance threshold of *p* < 0.005, we found three small connected components with decreased connectivity strength in the patients compared to normal controls (Figure 5(a)). Two were located in the right hemisphere, mainly consisting of several frontal regions and the thalamus. The remaining one involved three cingulate regions, connecting the bilateral cingulate gyri. However, the patients had two connected subnetworks with enhanced connectivity strength in the left hemisphere. The larger subnetwork with eight edges was located near the lesions, connecting six perisylvian regions and three subcortical regions. The smaller one with four edges mainly connected several limbic/paralimbic regions.

By contrast, the less strict initial *p* < 0.05 resulted in an extensive connected component with decreased connectivity strength in the patients relative to normal controls (Figure 5(b)). This altered subnetwork consisted of 22 edges and 22 nodes. The nodes involved mainly included a number of right perisylvian regions, two left frontal regions, and two subcortical regions.

### 3.5. Language Reorganization in the Patients

Functional lateralization of the frontal language area was evaluated for each patient. We found contralateral language reorganization in five (35.7%) patients with a mean LI of −0.50 ± 0.50, who were classified as atypically lateralized. The remaining nine (64.3%) patients had a mean LI of 0.87 ± 0.16 and were classified as typically lateralized. The global and nodal topological properties were compared between the two groups. We found that the clustering coefficient was significantly lower (*p* = 0.031) in atypically lateralized patients than in typically lateralized patients. However, no significant difference was found in other topological properties or in structural connectivity.

## 4. Discussion

The lacking capillary bed of an AVM causes a low-resistance condition and results in arteriovenous shunting of blood. The high-flow shunting diverts blood away from the surrounding brain tissue, leading to “arterial steal,” as well as raising the venous pressures, causing venous congestion [1, 4]. It has been revealed that such perfusion abnormalities may lead to chronic ischemic changes of the perifocal parenchyma, including neuron degeneration and gliosis [3, 33]. However, little is known about the long-range effects of AVMs on the white matter organization of the whole brain. Using DTI and graph theory methods, we investigated the whole-brain structural networks in a homogeneous series of patients with AVMs located in the presumed Broca's area. By comparing with normal controls, we identified the alterations in topological properties and network connectivity in the AVM patients.

### 4.1. Small-World Organization and Altered Global Properties

The small-world network organization of the human brain has been revealed in previous studies [34–36]. A small-world network can promote both segregated and integrated information processing, which is considered optimal compromise between regular and random networks [37]. In consistent with the past studies of stroke and tumors [20, 22], the current patient series exhibited small-world organization of brain structural networks, suggesting that the brain harboring AVMs supports efficient parallel information processing at a minimal cost. Although the small-worldness of the two groups was comparable, the patients exhibited significantly decreased clustering coefficient and increased characteristic path length. In graph theory, the clustering coefficient is a parameter reflecting the degree of concentration of connections and the capacity for segregated information processing, and a shorter characteristic path length ensures the rapid information integration between distant brain regions. We therefore suggest that the capacity for both segregated and integrated information processing is impaired in AVM patients. Additionally, we found a significant decrease in global efficiency in AVM patients, indicating a general decline of information transfer efficiency across the whole brain, which coincides with several previous studies of stroke patients [12, 19]. Nevertheless, the preserved small-worldness reflects the correction and resilience of the structural networks against pathological damages.

Thus, we suggest that the integrity and efficiency of the whole-brain structural networks are compromised in AVM patients, though there may be a degree of compensatory reorganization. In the current patient series, no one had presented with language dysfunction, and strict linguistic testing was not performed, so we failed to determine whether the disrupted topological organization is associated with declined language ability or occasional speech difficulties. Researches have proposed the disruption of structural network topology as a potential biomarker for a cognitive decline in several diseases [38–41]. Previous studies revealed high-incidence impaired cognitive performance in patients with AVMs, even the patients did not exhibit clinical symptoms [9, 42]. Future studies with thorough neuropsychological tests are needed to verify whether the impaired brain structural networks underlie the cognitive decline exhibited by AVM patients.

### 4.2. Altered Nodal Properties

In comparison with normal controls, AVM patients exhibited significantly reduced nodal connections in several regions. Most of the regions exhibiting decreased nodal strength coincided with those exhibiting decreased nodal efficiency, mainly located in the right perisylvian regions and the left occipital lobe. Furthermore, the local clustering coefficient was reduced in several right temporoparietal regions close to those regions with decreased nodal strength, suggesting weakened structural segregation of information processing within these neighboring regions. Interestingly, the brain regions with altered nodal properties were remote from the lesion sites, indicating the remote effects produced by AVMs. In a network analysis of stroke patients, the authors demonstrated reduced communicability not only in the lesioned hemisphere but also in the contralesional hemisphere, and they interpreted this remote alteration as the result of secondary white matter tract degeneration from the lesioned hemisphere [43]. We suppose that the reduced nodal connections of the left occipital lobe in the current series might be mainly attributed to the affected occipitofrontal fasciculus. De Baene et al. examined the functional network topology in patients with low-grade gliomas and found that the decrease in intramodular connectivity was coupled to the enhancement of distributed information integration in the contralesional hemisphere [13]. We infer that the reduced structural segregation in the right temporoparietal regions might reflect compensatory remodeling of the structural networks in the current patient series.

### 4.3. Altered Vulnerability and Hub Distribution

The vulnerability and betweenness centrality characterize the critical role of nodes from different aspects [44]. A higher vulnerability indicates that hypothetical removal of this node and its connections will result in a greater decline of the global efficiency. In this study, significantly increased vulnerability was found in the left supramarginal gyrus, suggesting that the role of this region was upregulated in these patients. The supramarginal gyrus of the dominant hemisphere is part of the perisylvian language network, playing an important role in various aspects of language processing such as syntactic and phonological processing [45]. This alteration might indicate a reorganization of brain structural networks for maintaining proper functioning of language in patients with Broca's area involved.

We used the level of betweenness centrality to identify the hubs of the structural networks. The betweenness centrality quantifies the role of nodes that act as bridges between the other nodes. Although there were no regions showing remarkable between-group difference in betweenness centrality, we found that the patients exhibited altered hub distribution compared to normal controls. Coinciding with the results of previous studies [34, 35], the precentral gyrus, thalamus, and several association areas were identified as hub regions in the current normal controls. Notably, the left superior temporal gyrus was identified as a hub in the normal controls but not in the patients. The posterior portion of the left superior temporal gyrus known as Wernicke's area is involved in language comprehension, connected directly with Broca's area through the arcuate fasciculus. The absence of this hub might be mainly attributed to the involvement of Broca's area and the arcuate fasciculus, reflecting a less left-lateralized language network in these patients. It is notable that the bilateral putamina appeared as hubs in the patients but not in normal controls, suggesting that the role of the putamen was enhanced in relaying information. The putamen has traditionally been associated with functions including motor control and reinforcement learning, but recent studies have demonstrated the participation of both the left and right putamen in language networks and various language functions, such as semantic, orthographic, and bilingual processing [46–48]. Thus, the increased importance of the putamen might be the evidence of structural network remodeling in demand for language functioning.

### 4.4. Altered Structural Connectivity

Although no significant difference of nodal properties was found in the brain regions near AVMs, we identified a subnetwork with enhanced structural connectivity involving the perifocal regions, which might reflect compensatory reorganization of the structural networks for maintaining language function in these patients. Additionally, we found another subnetwork with enhanced connectivity, mainly composed of the left amygdala and insulo-orbito-temporopolar component [49], suggesting the potential effects of AVMs on the limbic/paralimbic system.

Furthermore, we identified an extensive subnetwork with decreased structural connectivity in AVM patients compared with normal controls, which demonstrated weakened intraregional connectivity in the right perisylvian regions and weakened interhemispheric connectivity between Broca's area and the homologous regions of the right hemisphere. It is currently thought that the structural networks can predict and constrain the functional activity across the brain, and conversely, the functional activity can produce an effect on the structural networks [24, 50]. In this study, about one-third of patients exhibited varying degrees of contralateral language reorganization. We suppose that the reduced structural segregation of the right perisylvian regions might indicate the inhibition of the intrinsic functions of this area (e.g., spatial orienting), because of greater demand for information integration to compensate for the language function. Previously, researchers discussed the relationship between callosal connectivity and language lateralization and proposed the “inhibitory model” of the corpus callosum, where the interhemispheric connectivity hinders the functional interhemispheric integration and is negatively correlated with the functional activation of the right hemisphere [51, 52]. This theory could properly interpret the decreased structural connectivity between Broca's area and the right homologous regions in the current patient series. Controversially, since the conflicting “excitatory model” of the corpus callosum has been supported by other researchers [53, 54], the relationship between structural connectivity and language lateralization remains ambiguous. We suppose that under pathological conditions, the structural-functional coupling might be configured through a complicated way that involves a dynamic balance between inhibitory and excitatory effects, along with the interaction of segregated and integrated information processing. Future follow-up fMRI and DTI investigations are needed to examine the longitudinal changes of brain connectivity resulting from the long-range effects of AVMs.

### 4.5. Relevance of Language Reorganization

Functional reorganization has been frequently reported in AVM patients with eloquent areas involved [5, 7, 8], and therefore patients with unruptured lesions usually do not present with focal neurological dysfunction. In this study, none of the 14 patients with Broca's area directly involved had exhibited language deficits before participation. By using fMRI, we found that the incidence of atypical language lateralization was 35.7% in the patients, which was substantially higher than the reported incidence of 4% to 9% in healthy right-handed individuals [54–56]. Compared to the typically lateralized patients, the atypical lateralized patients exhibited significantly reduced global clustering coefficient, suggesting a reduced capacity for segregated information processing. Otherwise, we failed to identify significant difference in other topological properties or in structural connectivity. The lack of difference might reflect the indeterminate relationship between the structural networks and functional activities, although the results might be limited by the low number of patients.

### 4.6. Limitations

This is an exploratory study focusing on the whole-brain structural networks in patients with AVMs. Some issues should be addressed when interpreting the results: firstly, methodological issues. In this study, we used deterministic tractography to construct the structural network, but this algorithm failed to resolve the crossing fibers, which may result in a loss of existing fibers. The probabilistic tractography algorithm has been proposed to improve the network sensitivity, but it often has low specificity due to false positives [57]. In this study, we used the FreeSurfer automated method to define ROIs for its proven robustness in brain segmentation [28]. However, since there is no standard atlas for regional parcellation of the brain yet, the definition of network nodes was somewhat arbitrary, given that different nodes would produce different network properties. Secondly, although we tried to understand the changes of structural networks from the perspective of language function, we could not exactly determine which changes were detrimental, compensatory, or a combination of both. Thirdly, we lacked the data about subclinical cognitive states of the patients in this study. Nevertheless, our findings might provide a possible approach for evaluating the risk of cognitive decline taken by patients with AVMs. Fourthly, although we included a fairly homogeneous series of patients, the sample size was relatively small, which might limit the statistical power of the conclusions.

## 5. Conclusions

In conclusion, patients with AVMs had preserved small-worldness in brain structural networks, indicating brain resilience against pathologies. However, compared with the normal controls, AVM patients exhibited significantly decreased global efficiency and clustering coefficient, along with decreased corresponding nodal properties in some remote brain regions, such as the left occipital lobe and the right perisylvian regions. Furthermore, structural connectivity was reduced in the right perisylvian regions but was enhanced in the perifocal areas. The vulnerability of the left supramarginal gyrus was significantly increased, and the bilateral putamina were added as hubs in the AVM patients. The current study is the first to demonstrate the altered organization of brain structural networks in AVM patients, and the results provide evidence for the long-range effects of AVMs upon the whole-brain white matter networks. Our preliminary findings may contribute extra insights into the understanding of brain plasticity and pathological state in patients with AVMs.

## Figures and Tables

**Table 1:** Cortical and subcortical regions of interest defined in this study.

Regions	Abbreviation	Regions	Abbreviation
Bank of the superior temporal sulcus	BSTS	Posterior cingulate gyrus	PCG
Caudal anterior cingulate gyrus	cACG	Precentral gyrus	preC
Caudal middle frontal gyrus	cMFG	Precuneus	PCUN
Cuneus	CUN	Rostral anterior cingulate gyrus	rACG
Entorhinal cortex	ENT	Rostral middle frontal gyrus	rMFG
Fusiform gyrus	FUS	Superior frontal gyrus	SFG
Inferior parietal lobule	IPL	Superior parietal lobule	SPL
Inferior temporal gyrus	ITG	Superior temporal gyrus	STG
Isthmus cingulate gyrus	iCG	Supramarginal gyrus	SMAR
Lateral occipital gyrus	LOG	Frontal pole	FP
Lateral orbitofrontal gyrus	LOF	Temporal pole	TP
Lingual gyrus	LING	Transverse temporal gyrus	TT
Medial orbitofrontal gyrus	MOF	Insula	INS
Middle temporal gyrus	MTG	Thalamus	THAL
Parahippocampal gyrus	PARH	Putamen	PUT
Paracentral lobule	paraC	Pallidum	PALL
Pars opercularis	pOPER	Caudate	CAUD
Pars orbitalis	pORB	Hippocampus	HIPP
Pars triangularis	pTRI	Amygdala	AMYG
Pericalcarine cortex	periCAL	Nucleus accumbens	ACCU
Postcentral gyrus	postC		

Cortical regions were parcellated according to the Desikan-Killiany atlas, and subcortical regions according to Fischl et al.

**Table 2:** Basic characteristics of normal controls and patients.

Characteristics	Normal controls (*n* = 27)	Patients (*n* = 14)	*p*
Age (years)	25.0 ± 4.0	27.4 ± 7.0	0.087
Sex, female	15 (55.6%)	8 (57.1%)	0.923
Lesion diameter (cm)	—	3.6 ± 1.3	
Number of streamlines	7431.8 ± 1170.7	7241.1 ± 1067.9	0.527

Data are presented as mean ± standard deviation or number (%). *p* values refer to the Wilcoxon rank-sum test for age and number of streamlines and the chi-square test for sex.

**Table 3:** Hub regions of normal controls and patients.

Normal controls			Patients		
Regions	Mean *B*_*i*_	Mean *S*_*i*_	Regions	Mean *B*_*i*_	Mean *S*_*i*_
SPL R	0.1237	3.0204	SPL R	0.1424	2.1948
SPL L	0.1051	3.6080	SPL L	0.1193	2.5785
SFG L	0.0799	6.0365	SFG R	0.1006	4.6238
SFG R	0.0793	5.6838	THAL R	0.0924	5.2411
THAL R	0.0762	5.9460	preC L	0.0833	7.2224
THAL L	0.0700	6.1659	SFG L	0.0796	4.5024
PCUN L	0.0691	4.2896	THAL L	0.0646	6.4692
preC L	0.0675	6.9977	preC R	0.0582	4.8060
preC R	0.0604	7.1835	PUT R	0.0553	13.1882
PCUN R	0.0531	3.6712	PCUN R	0.0543	2.5686
STG L	0.0493	5.7540	PUT L	0.0539	16.4608
			PCUN L	0.0518	3.0028

*B*_*i*_ is the between centrality, and *S*_*i*_ is the nodal strength.

## Data Availability

The data used to support the findings of this study are available from the corresponding author upon request.

## References

[1] Choi J. H., Mohr J. P. (2005). Brain arteriovenous malformations in adults. *The Lancet Neurology*.

[2] Solomon R. A., Connolly E. S. (2017). Arteriovenous malformations of the brain. *The New England Journal of Medicine*.

[3] Attia W., Tada T., Hongo K. (2003). Microvascular pathological features of immediate perinidal parenchyma in cerebral arteriovenous malformations: giant bed capillaries. *Journal of Neurosurgery*.

[4] Kim D. J., Krings T. (2011). Whole-brain perfusion CT patterns of brain arteriovenous malformations: a pilot study in 18 patients. *American Journal of Neuroradiology*.

[5] Lee D. J., Pouratian N., Bookheimer S. Y., Martin N. A. (2010). Factors predicting language lateralization in patients with perisylvian vascular malformations. *Journal of Neurosurgery*.

[6] Deng X., Xu L., Zhang Y. (2016). Difference of language cortex reorganization between cerebral arteriovenous malformations, cavernous malformations, and gliomas: a functional MRI study. *Neurosurgical Review*.

[7] Alkadhi H., Kollias S. S., Crelier G. R., Golay X., Hepp-Reymond M. C., Valavanis A. (2000). Plasticity of the human motor cortex in patients with arteriovenous malformations: a functional MR imaging study. *American Journal of Neuroradiology*.

[8] Vikingstad E. M., Cao Y., Thomas A. J., Johnson A. F., Malik G. M., Welch K. M. A. (2000). Language hemispheric dominance in patients with congenital lesions of eloquent brain. *Neurosurgery*.

[9] de Souza Coelho D., Fernandes de Oliveira Santos B., Silva da Costa M. D. (2020). Cognitive performance in patients with cerebral arteriovenous malformation. *Journal of Neurosurgery*.

[10] Hart M. G., Romero-Garcia R., Price S. J., Suckling J. (2019). Global effects of focal brain tumors on functional complexity and network robustness: a prospective cohort study. *Neurosurgery*.

[11] Grefkes C., Fink G. R. (2011). Reorganization of cerebral networks after stroke: new insights from neuroimaging with connectivity approaches. *Brain*.

[12] Zhang J., Zhang Y., Wang L. (2017). Disrupted structural and functional connectivity networks in ischemic stroke patients. *Neuroscience*.

[13] De Baene W., Rutten G. J. M., Sitskoorn M. M. (2017). The temporal pattern of a lesion modulates the functional network topology of remote brain regions. *Neural Plasticity*.

[14] Rousseau P. N., La Piana R., Chai X. J., Chen J. K., Klein D., Tampieri D. (2019). Brain functional organization and structure in patients with arteriovenous malformations. *Neuroradiology*.

[15] Bullmore E., Sporns O. (2009). Complex brain networks: graph theoretical analysis of structural and functional systems. *Nature Reviews Neuroscience*.

[16] Salvador R., Suckling J., Coleman M. R., Pickard J. D., Menon D., Bullmore E. (2005). Neurophysiological architecture of functional magnetic resonance images of human brain. *Cerebral Cortex*.

[17] Tinaz S., Lauro P. M., Ghosh P., Lungu C., Horovitz S. G. (2017). Changes in functional organization and white matter integrity in the connectome in Parkinson's disease. *NeuroImage: Clinical*.

[18] Bai F., Shu N., Yuan Y. (2012). Topologically convergent and divergent structural connectivity patterns between patients with remitted geriatric depression and amnestic mild cognitive impairment. *The Journal of Neuroscience*.

[19] Sang L., Liu C., Wang L. (2020). Disrupted brain structural connectivity network in subcortical ischemic vascular cognitive impairment with no dementia. *Frontiers in Aging Neuroscience*.

[20] Shi L., Wang D., Chu W. C. W. (2013). Abnormal organization of white matter network in patients with no dementia after ischemic stroke. *PLoS One*.

[21] Bonilha L., Gleichgerrcht E., Nesland T., Rorden C., Fridriksson J. (2016). Success of anomia treatment in aphasia is associated with preserved architecture of global and left temporal lobe structural networks. *Neurorehabilitation and Neural Repair*.

[22] Yu Z., Tao L., Qian Z. (2016). Altered brain anatomical networks and disturbed connection density in brain tumor patients revealed by diffusion tensor tractography. *International Journal of Computer Assisted Radiology and Surgery*.

[23] van den Heuvel M., Mandl R., Luigjes J., Hulshoff Pol H. (2008). Microstructural organization of the cingulum tract and the level of default mode functional connectivity. *The Journal of Neuroscience*.

[24] Honey C. J., Sporns O., Cammoun L. (2009). Predicting human resting-state functional connectivity from structural connectivity. *Proceedings of the National Academy of Sciences of the United States of America*.

[25] Nachev P., Coulthard E., Jager H. R., Kennard C., Husain M. (2008). Enantiomorphic normalization of focally lesioned brains. *NeuroImage*.

[26] Rorden C., Bonilha L., Fridriksson J., Bender B., Karnath H. O. (2012). Age-specific CT and MRI templates for spatial normalization. *NeuroImage*.

[27] Solodkin A., Hasson U., Siugzdaite R. (2010). Virtual brain transplantation (VBT): a method for accurate image registration and parcellation in large cortical stroke. *Archives Italiennes de Biologie*.

[28] Fischl B. (2012). FreeSurfer. *Neuroimage*.

[29] Fischl B., Salat D. H., Busa E. (2002). Whole brain segmentation: automated labeling of neuroanatomical structures in the human brain. *Neuron*.

[30] Bradshaw A. R., Thompson P. A., Wilson A. C., Bishop D. V. M., Woodhead Z. V. J. (2017). Measuring language lateralisation with different language tasks: a systematic review. *PeerJ*.

[31] Ashburner J., Friston K. J. (2005). Unified segmentation. *NeuroImage*.

[32] Zalesky A., Fornito A., Bullmore E. T. (2010). Network-based statistic: identifying differences in brain networks. *NeuroImage*.

[33] Essig M., Wenz F., Schoenberg S. O., Debus J., Knopp M. V., van Kaick G. (2000). Arteriovenous malformations: assessment of gliotic and ischemic changes with fluid-attenuated inversion-recovery MRI. *Investigative Radiology*.

[34] Iturria-Medina Y., Sotero R. C., Canales-Rodriguez E. J., Aleman-Gomez Y., Melie-Garcia L. (2008). Studying the human brain anatomical network via diffusion-weighted MRI and graph theory. *NeuroImage*.

[35] Achard S., Salvador R., Whitcher B., Suckling J., Bullmore E. (2006). A resilient, low-frequency, small-world human brain functional network with highly connected association cortical hubs. *The Journal of Neuroscience*.

[36] He Y., Chen Z. J., Evans A. C. (2007). Small-world anatomical networks in the human brain revealed by cortical thickness from MRI. *Cerebral Cortex*.

[37] Watts D. J., Strogatz S. H. (1998). Collective dynamics of 'small-world' networks. *Nature*.

[38] Reijmer Y. D., Leemans A., Caeyenberghs K. (2013). Disruption of cerebral networks and cognitive impairment in Alzheimer disease. *Neurology*.

[39] Du J., Wang Y., Zhi N. (2019). Structural brain network measures are superior to vascular burden scores in predicting early cognitive impairment in post stroke patients with small vessel disease. *NeuroImage: Clinical*.

[40] Hawkins R., Shatil A. S., Lee L. (2020). Reduced global efficiency and random network features in patients with relapsing-remitting multiple sclerosis with cognitive impairment. *American Journal of Neuroradiology*.

[41] Berlot R., Metzler-Baddeley C., Ikram M. A., Jones D. K., O'Sullivan M. J. (2016). Global efficiency of structural networks mediates cognitive control in mild cognitive impairment. *Frontiers in Aging Neuroscience*.

[42] Lazar R. M., Connaire K., Marshall R. S. (1999). Developmental deficits in adult patients with arteriovenous malformations. *Archives of Neurology*.

[43] Crofts J. J., Higham D. J., Bosnell R. (2011). Network analysis detects changes in the contralesional hemisphere following stroke. *NeuroImage*.

[44] Rubinov M., Sporns O. (2010). Complex network measures of brain connectivity: uses and interpretations. *NeuroImage*.

[45] Binkofski F. C., Klann J., Caspers S., Hickok G., Small S. L. (2016). Chapter 4 - on the neuroanatomy and functional role of the inferior parietal lobule and intraparietal sulcus. *Neurobiology of Language*.

[46] Mitsuhashi T., Sugano H., Asano K. (2020). Functional MRI and structural connectome analysis of language networks in Japanese-English bilinguals. *Neuroscience*.

[47] Vinas-Guasch N., Wu Y. J. (2017). The role of the putamen in language: a meta-analytic connectivity modeling study. *Brain Structure & Function*.

[48] Abutalebi J., Rosa P. A. D., Castro Gonzaga A. K., Keim R., Costa A., Perani D. (2013). The role of the left putamen in multilingual language production. *Brain and Language*.

[49] Mesulam M. M., Mufson E. J. (1982). Insula of the old world monkey. I. Architectonics in the insulo-orbito-temporal component of the paralimbic brain. *The Journal of Comparative Neurology*.

[50] Hagmann P., Sporns O., Madan N. (2010). White matter maturation reshapes structural connectivity in the late developing human brain. *Proceedings of the National Academy of Sciences of the United States of America*.

[51] Josse G., Seghier M. L., Kherif F., Price C. J. (2008). Explaining function with anatomy: language lateralization and corpus callosum size. *The Journal of Neuroscience*.

[52] Putnam M. C., Wig G. S., Grafton S. T., Kelley W. M., Gazzaniga M. S. (2008). Structural organization of the corpus callosum predicts the extent and impact of cortical activity in the nondominant hemisphere. *The Journal of Neuroscience*.

[53] van der Knaap L. J., van der Ham I. J. (2011). How does the corpus callosum mediate interhemispheric transfer? A review. *Behavioural Brain Research*.

[54] Haberling I. S., Badzakova-Trajkov G., Corballis M. C. (2011). Callosal tracts and patterns of hemispheric dominance: a combined fMRI and DTI study. *NeuroImage*.

[55] Isaacs K. L., Barr W. B., Nelson P. K., Devinsky O. (2006). Degree of handedness and cerebral dominance. *Neurology*.

[56] Knecht S., Deppe M., Dräger B. (2000). Language lateralization in healthy right-handers. *Brain*.

[57] Zalesky A., Fornito A., Cocchi L., Gollo L. L., van den Heuvel M. P., Breakspear M. (2016). Connectome sensitivity or specificity: which is more important?. *NeuroImage*.

